# Diabetic macular edema treated with intravitreal aflibercept injection after treatment with other anti-VEGF agents (SWAP-TWO study): 6-month interim analysis

**DOI:** 10.1186/s40942-019-0167-x

**Published:** 2019-07-23

**Authors:** Amy S. Babiuch, Thais F. Conti, Felipe F. Conti, Fabiana Q. Silva, Aleksandra Rachitskaya, Alex Yuan, Rishi P. Singh

**Affiliations:** 1 Cole Eye Institute, Cleveland Clinic, 9500 Euclid Avenue, i32, Cleveland, OH 44195 USA; 2 Center for Ophthalmic Bioinformatics, Cole Eye Institute, Cleveland Clinic, Cleveland, USA; 3 Federal University of São Paulo, São Paulo, Brazil; 4 Case Western Reserve University, Cleveland, USA

## Abstract

**Background:**

Diabetic macular edema (DME) is an important cause of vision loss and despite the anatomical and functional improvement achieved with treatment, there are reports of persistent DME regardless of continuous anti-VEGF therapy. The purpose of this study is to examine the effect of patients with DME previously treated with other anti-VEGF agents who are transitioned to intravitreal aflibercept (IAI) on a fixed dosing regimen.

**Methods:**

This prospective study included 20 patients presenting with DME with a history of previous anti-VEGF treatment with ranibizumab or bevacizumab. Patients received a 2 mg (0.05 mL) IAI every 4 weeks until no evidence of fluid by optical coherence tomography (OCT) followed by a fixed dosing schedule of 2 mg IAI once every 8 weeks through 24 months. There was a pre-planned interim analysis of the mean absolute change from baseline central foveal thickness at month 6 as measured by OCT. Secondary outcomes included mean change from baseline in ETDRS visual acuity and anatomic parameters. Optical Coherence tomography angiography (OCTA) capillary perfusion density (CPD) after transitioning to IAI therapy were also reported.

**Results:**

Average central subfield thickness on OCT at baseline was 419.7 ± 92.0 and improved to 303.8 ± 73.1 at 6-months (*p* < 0.001). At 6 months after IAI treatment, BCVA increased + 1.5 letters from baseline (*p* = 0.38). OCTA CPD analysis revealed significant increase from baseline in the foveal avascular zone in non-proliferative diabetic retinopathy group (*p* = 0.02).

**Conclusions:**

Patients with prior anti-VEGF therapy who were transitioned to IAI therapy revealed significant anatomic improvements through 6 months.

*Trial registration* Treatment of Diabetic Macular Edema With Aflibercept in Subjects Previously Treated With Ranibizumab or Bevacizumab (SwapTwo), Trial registration number: NCT02559180. Date of registration: September 24, 2015.https://clinicaltrials.gov/ct2/show/NCT02559180

## Introduction

Diabetes mellitus is one of the most common chronic diseases and continues to rise in numbers and significance. Late estimates show a global prevalence of 382 million patients, expected to rise to 592 million by 2035 [1]. Diabetic macular edema (DME) is an important cause of vision loss in diabetic patients present in 20% of patients with younger onset versus approximately 40% in older onset diabetes [2, 3].

Anti-vascular endothelial growth factor (VEGF) has become the first-line treatment for DME [4]. Three VEGF inhibitors are commonly used: aflibercept (Eylea, Regeneron Pharmaceuticals, Tarrytown, NY, USA) and ranibizumab (Lucentis, Genentech, South San Francisco, California, USA) are Food and Drug Administration (FDA) approved treatment for DME, while bevacizumab (Avastin, Genentech, South San Francisco, California, USA) is used off-label. Despite the anatomical and functional improvement achieved with treatment, there are reports of persistent DME regardless of continuous anti-VEGF therapy. RISE and RIDE trials reported that, after 24 months of ranibizumab therapy, 27–46% of eyes had vision of 20/40 or worse and 19–26% of eyes still had central subfield thickness (CST) greater than 250 µm [5]. Protocol I reported persistent DME, defined as never having CST below 250 µm, in around 40% of eyes receiving monthly ranibizumab after 6 months [6]. Therefore, switching to a drug with different VEGF affinity and that can block other cytokine pathways maximizing therapeutic potential has become a common practice among retinal specialists [7]. Most physicians (77.5%) consider switching anti-VEGF agent due inadequate response after three to six injections, and the majority (59%) will have noticed visual improvements after the switching [4]. However, there is a lack of well-designed, prospective, randomized clinical trials assessing the efficacy of switching anti-VEGF on DME outcomes.

DRCR Network Protocol T, a multicenter, randomized clinical trial comparing aflibercept, bevacizumab, and ranibizumab in patients with center-involved DME, reported better visual and anatomical outcomes from baseline to 1 year with aflibercept, especially in patients with visual acuity ≤ 20/50 [8]. However, Protocol T used a rigid inclusion criterion which required patients not to have anti-VEGF therapy for a minimum of 12 months prior to entry. While most studies employ a fixed monthly dosing schedule, the majority of retina specialists do not treat using a monthly dosing pattern, instead less burdensome treatment paradigms, such as as-needed (pro ne rata, PRN) or treat-and-extend (TAE) dosing are used. However, the extension studies of RESTORE, RISE and RIDE trials demonstrate visual acuity (VA) decline in subjects transitioned to a more flexible treatment from a fixed dosing scheme [9, 10].

This study aims to evaluate the effects of switching patients with DME previously treated with other anti-VEGF agents to intravitreal aflibercept (IAI), and further placing them on a fixed dosing regimen.

## Methods

The SWAP-TWO Study is a prospective, interventional, single arm study performed at the Cole Eye Institute, Cleveland, Ohio, USA. The study received approval from the Cleveland Clinic Investigational Review Board (IRB), and all study-related procedures were performed in accordance with good clinical practice (International Conference on Harmonization of Technical Requirements for Registration of Pharmaceuticals for Human Use (ICH) E6), applicable FDA regulations, and the Health Insurance Portability and Accountability Act. All patients signed an informed consent for their participation in the study.

### Participants

This prospective study enrolled 20 eyes of 20 patients between December 2015 and August 2017. Eligible participants were aged ≥ 18 years with foveal-involving retinal edema secondary to diabetic retinopathy (DR) based on investigator review of clinical exam and spectral-domain optical coherence tomography (SD-OCT) with CST value of 325 µm, Early Treatment Diabetic Retinopathy Study (ETDRS) best-corrected visual acuity (BCVA) of 80 (20/25) to 20 (20/400) in the study eye, and history of previous treatment with bevacizumab or ranibizumab with at least 4 previous injections in the last 6 months.

Patients were excluded if they had any prior or concomitant therapy with another investigational agent to treat DME in the studied eye, history of vitrectomy or panretinal photocoagulation in the study eye within 3 months, or previous intravitreal anti-VEGF therapy in the study eye within 30 days of enrollment. Patients with history of IAI were not allowed in the study. Prior systemic anti-VEGF therapy, investigational or FDA-approved, was only allowed up to 3 months prior to first dose. Additionally, individuals were excluded if they had any of the following concomitant ocular diseases at baseline evaluation: uncontrolled glaucoma, active intra-ocular or periocular infection in either eye, other causes of macular edema including pathologic myopia (spherical equivalent of –8 diopters or more negative, or axial length of 25 mm or more), presumed ocular histoplasmosis syndrome or any history of uveites, angioid streaks, choroidal rupture, choroidal neovascularization, age-related macular degeneration or multifocal choroiditis in the study eye. Epiretinal membranes were not considered an exclusion. Only one eye per subject was enrolled in the study. For patients who met eligibility criteria in both eyes, the investigator and patient designated the study eye. If a non-study eye required treatment for DME at study entry or during the subject’s participation in the study, the fellow eye could receive IAI, but it was not considered as an additional study eye. The frequency of fellow eye treatment was based on investigator discretion.

### Visits and assessments

**Fig. 1:**
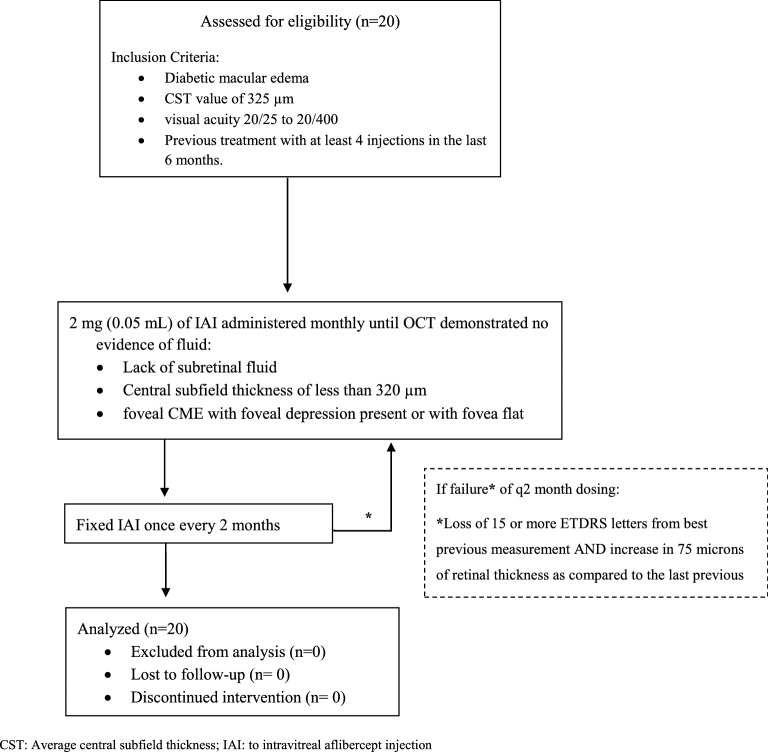
Flow chart

Patients were given 2 mg (0.05 mL) of IAI administered monthly until OCT demonstrated no evidence of fluid (defined as lack of subretinal fluid; central subfield thickness of less than 320 µm; extrafoveal cystoid macular edema (CME); or foveal CME with foveal depression present or with fovea flat) followed by fixed IAI once every 2 months (Fig. 1). IAI was supplied by Regeneron Pharmaceuticals, and was administered using the standard aseptic.

Study visits were scheduled every 28 ± 7 days. At each visit, the visual functions of both the eyes were assessed using the ETDRS chart (M&S Systems) and protocol visual acuity measurement consisting of BCVA testing and a forced choice paradigm [11]. A comprehensive eye examination and SD-OCT scanning were performed. The scanning protocol consisted of fast macular thickness maps as well as high definition 6.0 mm linear scans centered on the fovea using the Cirrus SD-OCT (Zeiss, San Leandro, California, USA) and a 3 × 3 mm (9 mm^2^) en face retinal map for vascular analysis using AngioVue Avanti RTVue XR (Optovue, Inc., Fremont, CA, USA).

Quantitative CPD analysis was performed with ReVue software version 2017.1.0.129 (Optovue, Inc, Fremont, CA). The built-in software calculates the capillary perfusion density (CPD) by computing the percentage area occupied by detected optical coherence tomography angiography (OCTA) vasculature. Vessels pixels area is ‘true’ while background/noise is ‘false’. This binary mask is then used to generate a 2D local density map and automatically calculated density values in grid sectors. Auto-segmentation was used to define anatomical borders for CPD analysis and for FAZ area demarcation. Manual corrections were made if any scan errors were identified.

Additional treatment with IAI could be applied if the subject experienced loss of 15 or more ETDRS letters from best previous measurement or increase in 75 µm of CST as compared to the best visit.

### Outcome measures

SD-OCT central subfield thickness, ETDRS BCVA, and OCTA capillary perfusion density studies were monitored after enrollment, and CST data was also collected retrospectively for the 6 month period prior to enrollment. The primary outcome of the study was defined as the mean absolute change from baseline central foveal thickness at month 12 with pre-planned interim analysis as measured by SD-OCT (defined as the average thickness within the central 1 mm subfield) at month 6. Secondary outcomes included the efficacy of treatment outcomes by improvements in ETDRS BCVA from baseline, perfusion changes in OCTA before and after therapy, as well as safety and tolerability of IAI therapy by monitoring adverse events (AEs).

### Safety analyses

Safety evaluations included ocular and non-ocular events reported by patients during or between study visits. AEs could also be detected through assessment and were recorded in case report forms. AEs were categorized according to severity (mild, moderate or severe) and relationship to study drug (related/not related).

### Statistical methods

Measures were summarized using means, standard deviation (SD), median and range. Normality of measures was evaluated using the Shapiro–Wilk test. Since data were mostly normally distributed, comparisons with and between groups were performed using two-sided paired t-tests. Where appropriate, sensitivity analyses using nonparametric Wilcoxon signed rank tests were also performed. Analyses were performed using SAS software (V.9.2; Cary, North Carolina, USA). A significance level of 0.05 was assumed for all tests.

## Results

Data was collected on 20 unique patients from baseline to 6 months. The mean age was 63.7 (range, 45–78) years, and 13 patients (65%) were female (Table 1). The average number of anti-VEGF treatments before transitioning was 4.25 (4–6) injections in the last 6 months with an average washout time of 44.4 days (± 21.2); 95% were injected with bevacizumab and 5% with ranibizumab prior to enrollment. No patient received more than one anti-VEGF drug prior to study enrollment. The mean baseline BCVA was 70 ± 7.2 (60–81) letters (≅ 20/40). The mean CST upon study entry was 419.7 ± 92 (328–585) µm. At baseline, 9 eyes (45%) were classified as mild/moderate non-proliferative diabetic retinopathy (NPDR), 5 (25%) severe NPDR, and 6 (30%) non-active proliferative diabetic retinopathy (PDR).

**Table 1:** Baseline demographics and ocular characteristics

Demographics	Baseline
Eyes^a^ (right:left)	20 (11:9)
Average age at screening	63.7 (45–78)
Gender (female:male)	13:7
Average prior injections^b^	4.25
ETDRS scores: average (range)	
Study eye	69.95 (60–81)
Fellow eye	73.65 (37–85)
Diabetic retinopathy severity	
Mild	0
Moderate	9
Severe	5
PDR	6
OCT values	
Study eye	
CST [Mean (range)]	419.7 (328–585)
Cube volume [Mean (range)]	11.55 (9.1–13.9)
Cube average thickness [Mean (range)]	320.7 (253–386)
Fellow eye	
CST [Mean (range)]	300.40 (181–432)
Cube volume [Mean (range)]	10.81 (8.8–12.8)
Cube average thickness [Mean (range)]	300.20 (246–354)

^a^Only one eye per patient, ^b^on the 6 month prior enrollment

*CST* central subfield thickness, *ETDRS* early treatment diabetic retinopathy study, *OCT* optical coherence tomography

### Treatment frequency

During the study, the mean number of IAI treatments were 5.25 (4–6), over an average 5.3 (4–6) visits. An average of 2.15 (0–6) fellow eye injections were given in 65% patients. At month 6, 11 (55%) patients still required monthly treatment, while 9 (45%) were able to receive IAI every other month after an average of 3.3 injections prior change.

### Anatomic outcomes

Table 2 demonstrates the differences between study variables at 6 months prior to enrollment, baseline, and month 6. CST, Cube Volume (CV), and Cube Average Thickness (CAT) were all significantly lower at month 6 (*p* < 0.001 for all variables) compared to baseline. CST improved from 419.7 ± 92.0 (328–585) µm at baseline to 303.8 ± 73.1 (198–485) µm at month 6 (27.63% reduction). CST measurements in the 6 month period prior to the baseline enrollment and drug switch were not statistically significant. Mean baseline CST at the visit 6 month visit prior to enrollment was 420.8 ± 100.5 and at baseline enrollment prior to drug switch was 419.7 ± 92.0 (*p* = 0.99).

**Table 2:** Differences between study variables from 6 months prior to enrollment, baseline and month 6

Factor	6 Months prior to enrollment	Baseline	Month 6	95% CI for changes	6 Months prior to enrollment to baseline	Baseline to month 6
	[Mean ± SD]	[Mean ± SD]	[Mean ± SD]		*P* value	*P* value
ETDRS BCVA	70.1 ± 7.7	70.0 ± 7.2	71.5 ± 8.9	1.55 (− 2.08, 5.18)	0.95	0.38
Central subfield thickness (µm)	420.8 ± 100.5	419.7 ± 92.0	303.8 ± 73.1	− 116.0 (− 150.8, − 81.15)	0.99	**< ** ***0.001***
Cube volume (mm^3^)	N/A	11.5 ± 1.4	10.7 ± 1.2	− 0.86 (− 1.06, − 0.65)	N/A	**< ** ***0.001***
Cube average thickness (µm)	N/A	320.7 ± 38.6	297.2 ± 33.1	− 23.50 (− 29.36, − 17.64)	N/A	**< ** ***0.001***
Foveal avascular zone (mm^2^)	N/A	0.31 ± 0.13	0.33 ± 0.11^a^	− 0.03(− 0.11, 0.05)	N/A	0.47

Statistically significant values are in bold italic

*ETDRS* early treatment diabetic retinopaty study, *BCVA* best corrected visual acuity, *CI* confidence interval

^a^n = 18

**Fig. 2:**
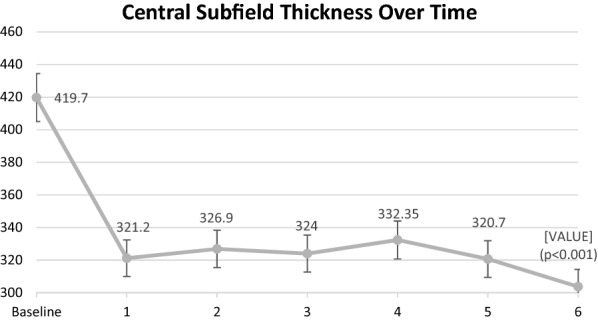
Visit-to-visit change in central subfield thickness

**Fig. 3:**
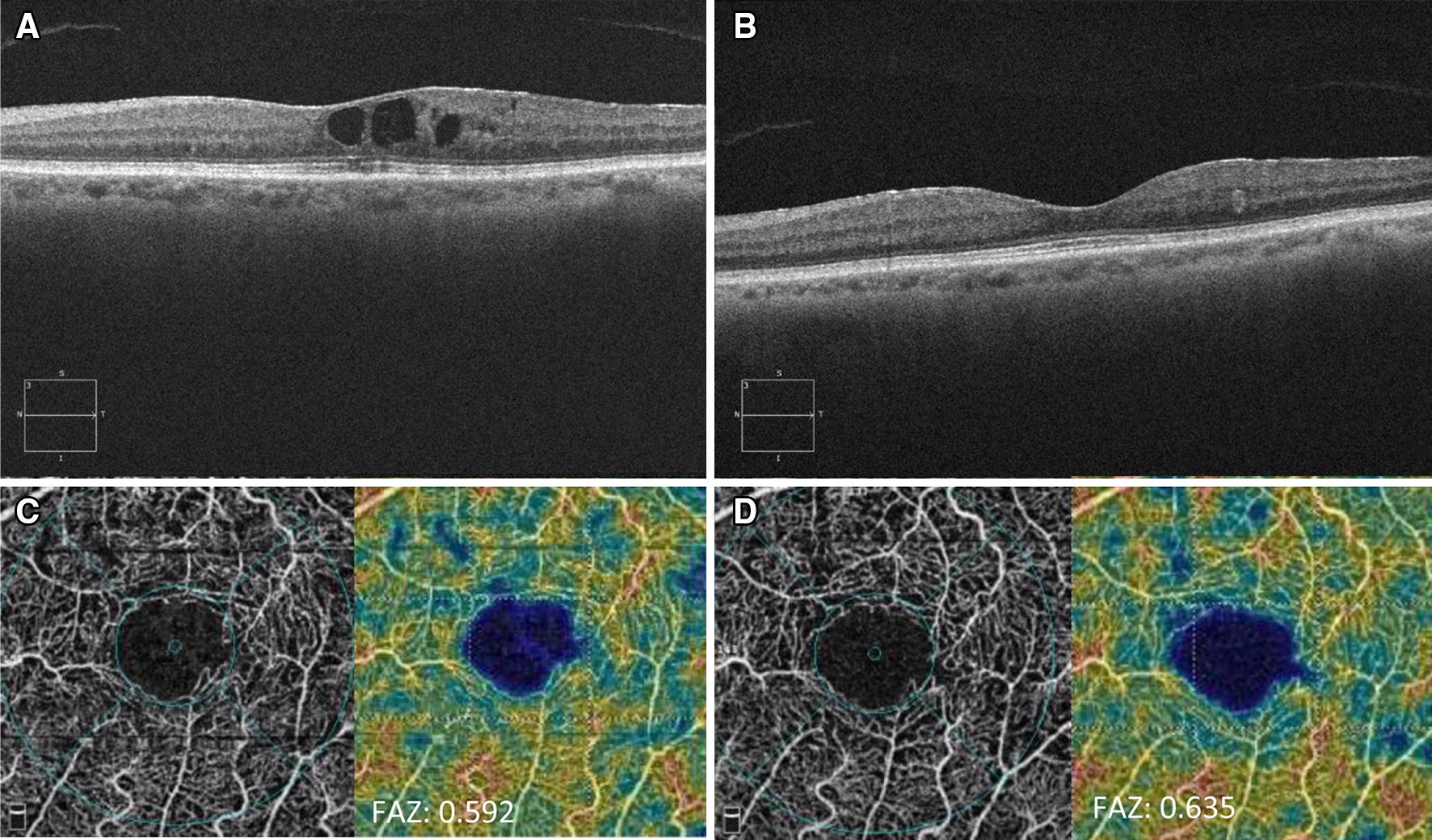
Optical coherence tomography and optical coherence tomography angiography of a patient with epiretinal membrane at baseline. **A** OCT from a patient with epiretinal membrane at baseline. **B** Patient 6 month after switching to IAI. **C** Example of En Face OCT and full retina capillary perfusion density analysis from same patient presented with ERM at baseline. **D** Example of En Face OCT and full retina capillary perfusion density analysis 6 month after switching to IAI

Measurements of CST at consecutive study visits are outlined in Fig. 2. Each subsequent visit had statistically significant improvement in CST from baseline (*p* < 0.001 for all time-point comparisons). The average CV improved from 11.5 ± 1.4 (9.1–13.9) to 10.7 ± 1.2 (8.8–12.8) mm^3^ at month 6 (7.4% decrease). The mean CAT improved from 320.7 ± 38.6 (253–386) to 297.2 ± 33.1 (234 – 368) µm at month 6 (7.3% reduction). Throughout the study period, no change in DR severity was observed. Two patients had epiretinal membrane at baseline and no visually change was seen at 6 month (Fig. 3).

Foveal avascular zone (FAZ) area was 0.31 mm^2^ at baseline and increased to 0.33 mm^2^ at 6 months. However, change from baseline to month 6 was not statistically significant (*p* = 0.47). When broken down by RD severity, the NPDR group (n = 15) showed an area of 0.27 mm^2^ at baseline, which increased to 0.32 mm^2^ at month 6 (*p* = 0.02). NPDR group also showed a significant improvement in CST (− 88.1 µm, *p* = 0.004). PDR group (n = 5) presented an area of 0.34 mm^2^ at baseline with a non-significant increase to 0.35 mm^2^ at month 6 (*p* = 0.7). In contrast, CST had statistically significant improvement from baseline in PDR group (− 109.8 µm, *p* = 0.01). No significant changes to retina density were observed between baseline and 6-months (46.8 ± 5.4 vs. 45.3 ± 5.2, *p* = 0.37).

### Visual acuity

**Fig. 4:**
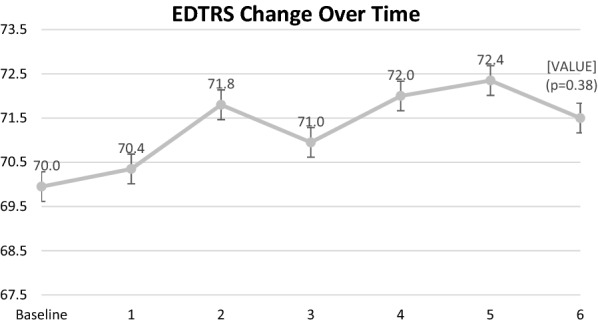
Visit-to-visit change in best correct visual acuity. *ETDRS* early treatment diabetic retinopathy study

**Fig. 5:**
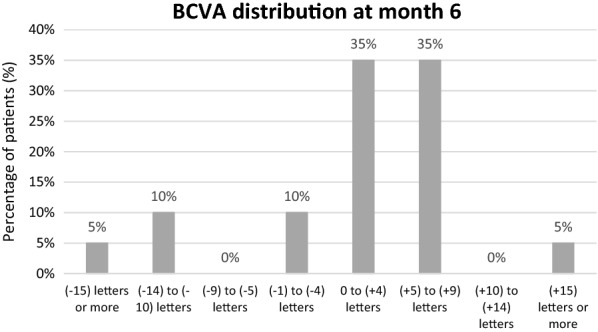
Visual acuity changes in patients noted by month 6. *BCVA* best-corrected visual acuity

Mean BCVA at the 6 month visit prior to enrollment and drug switch was 70.1 ± 7.7 and at baseline was 70.0 ± 7.2 (*p* = 0.95). BCVA increased minimally between the baseline visit and 6 months, [70.0 ± 7.2 (60–81) to 71.5 ± 8.9 (54–83) letters], but this change was not statistically significant (*p* = 0.38). BCVA measurements at consecutive visits are outlined in Fig. 4. At baseline, 65% (n = 13) patients were 20/40 or better, 35% (n = 7) patients were 20/50 or worse, and no patients were 20/200 or worse. By the end of month 6, 60% (12) patients were 20/40 or better, 40% (8) patients were 20/50 or worse, and no patients were 20/200 or worse. Figure 5 demonstrates the VA changes in patients noted by month 6.

### Safety

Three serious AEs were registered during the follow-up. At month 6, one study subject experienced chest pain, and cardiac catheterization was performed. Another was hospitalized for bilateral leg cellulitis (Month 6), and a third patient was hospitalized for dehydration, hypertension, and hyperglycemia (Month 2). No serious ocular AEs, including endophthalmitis, uveitis, retinal detachment, retinal pigment epithelial tears, submacular hemorrhage, or sustained elevated intraocular pressure requiring intervention were observed.

## Discussion

Results of this study demonstrated that, after switching to IAI, significant anatomical improvements were achieved. Several studies have assessed switching anti-VEGF in DME [12–25]. Retrospective studies with similar follow-up periods have reported significant CST improvement without BCVA gain [12, 20, 26]. The statistically significant anatomic improvement demonstrated after converting to IAI may be explained by several factors. IAI has been shown to bind VEGF with affinities 94 times stronger than ranibizumab, and 119 times greater than bevacizumab [27]. Additionally, IAI also binds VEGF-B and placental growth factor (PLGF) [27]. The concentration of PLGF plays a role in DR pathogenesis through increased levels of VEGF, and the activation of protein kinase which affects the blood retinal barrier [28]. The achievement of superior outcomes after conversion switching may not necessarily reflect only the drug superiority but also the benefit of a fixed-dosing paradigm in an FDA-approved manner over the possible undertreatment of variable-dosing regimens [9].

In contrast, Lim et al. reported distinct functional outcomes when assessing anti-VEGF switching to IAI from ranibizumab and/or bevacizumab in 21 eyes [16]. In their study, BCVA gain was significant at 5 months after switching, but not all patients included had persistent DME at baseline. Mira et al. also reported anatomical and functional gains 3 months following the transition to another anti-VEGF agent [19]. However, baseline BCVA in their study was 0.7 logmar (≅ . 20/100) which is considerably worse than the average baseline BCVA in this report, and in the general population. Since poor baseline BCVA is an important predictor of functional improvement in DME treatment that is probably a reason for the different outcomes reported [29]. Prospective data is also available and most studies reported no visual improvement despite a significant universal reduction in the CST [15, 17, 21]. A pos-hoc analysis of VIVID/VISTA trials reported that the rise in visual acuity is gradual, and visual acuity peak is only established after 6–9 months of treatment or longer [30].

This study reports no difference in FAZ area after 6 months treatment with IAI. Outcomes of other reports on effects of chronic anti-VEGF therapy on FAZ area are contradictory. Evidence regarding the ability of anti-VEGF agents to halt the progression of ischemic change is inconclusive. Some reports have asserted that continued anti-VEGF therapy can decrease the progression of the ischemic damage in patients with retinal microangiopathies [31, 32], while others have not notified any alterations [33, 34], and yet others have noted worsening of capillary drop out [35–38]. Even if anti-VEGF agents do reduce ischemic injury in the foveal area, this outcome might reach a ceiling effect with continuous treatment, and hence could explain why this study failed to show significant FAZ area changes in a patient population undergoing chronic anti-VEGF therapy.

Drawbacks of previous studies on the switching of patients to IAI include their retrospective design, variable follow up time, and a lack of protocol defined ETDRS BCVA and anatomic assessments. This prospective study allowed for entry criteria seen in clinical practice, and consistent evaluation of anatomical and visual outcomes. The limitations of this study include the small sample size, and lack of standardized treatment regimen prior to entry with other anti-VEGF drugs in addition to the short follow-up time.

Overall, this study begins to offer useful clinical insights into switching anti-VEGF medications followed by extended treatment interval IAI. Improved anatomic outcomes were achieved in this interim 6 month analysis, although visual outcomes were not statistically significant. The 12-month results of this study will be helpful in determining whether vision does indeed improve with continued treatment, and if fixed-dosing at a longer interval once macular fluid is resolved can sustain these positive effects.

## Data Availability

The datasets used and/or analyzed during the current study are available from the corresponding author on reasonable request.

## Abbreviations

VEGF: vascular endothelial growth factor
DME: diabetic macular edema
FDA: food and drug administration
CST: central subfield thickness
PRN: pro ne rata
TAE: treat-and-extend
VA: visual acuity
IAI: intravitreal aflibercept
IRB: investigational review board
DR: diabetic retinopathy
SD-OCT: spectral-domain optical coherence tomography
ETDRS: early treatment diabetic retinopathy study
BCVA: best-corrected visual acuity
CME: cystoid macular edema
OCTA: optical coherence tomography angiography
CPD: capillary perfusion density
AEs: adverse events
SD: standard deviation
NPDR: non-proliferative diabetic retinopathy
CV: cube volume
CAT: cube average thickness
FAZ: foveal avascular zone
PLGF: placental growth factor
